# Evaluation of the *MC4R* gene across eMERGE network identifies many unreported obesity-associated variants

**DOI:** 10.1038/s41366-020-00675-4

**Published:** 2020-09-20

**Authors:** Bahram Namjou, Ian B. Stanaway, Todd Lingren, Frank D. Mentch, Barbara Benoit, Ozan Dikilitas, Xinnan Niu, Ning Shang, Ashley H. Shoemaker, David J. Carey, Tooraj Mirshahi, Rajbir Singh, Jordan G. Nestor, Hakon Hakonarson, Joshua C. Denny, David R. Crosslin, Gail P. Jarvik, Iftikhar J. Kullo, Marc S. Williams, The eMERGE Network, John B. Harley

**Affiliations:** 1 Center for Autoimmune Genomics and Etiology, Cincinnati Children’s Hospital Medical Center (CCHMC), Cincinnati, OH USA; 2 College of Medicine, University of Cincinnati, Cincinnati, OH USA; 3 Department of Biomedical Informatics Medical Education, School of Medicine, University of Washington, Seattle, WA USA; 4 Division of Biomedical Informatics, Cincinnati Children’s Hospital Medical Center, Cincinnati, OH USA; 5 Center for Applied Genomics, Children’s Hospital of Philadelphia, Philadelphia, PA USA; 6 Research Information Science and Computing, Partners HealthCare, Somerville, MA USA; 7 Department of Cardiovascular Medicine, Mayo Clinic, Rochester, MN USA; 8 Departments of Biomedical Informatics and Medicine, Vanderbilt University, Nashville, TN USA; 9 Department of Biomedical Informatics, Columbia University, New York, NY USA; 10 Department of Pediatrics, Division of Endocrinology and Diabetes, Vanderbilt University Medical Center, Nashville, TN USA; 11 Department of Molecular and Functional Genomics, Geisinger, Danville, PA USA; 12 Meharry Medical College, Nashville, TN USA; 13 Department of Medicine, Division of Nephrology, Columbia University, New York, NY USA; 14 Department of Pediatrics, Perelman School of Medicine, University of Pennsylvania, Philadelphia, PA USA; 15 Department of Medicine (Medical Genetics), University of Washington Medical Center, Seattle, WA USA; 16 Department Genome Sciences, University of Washington Medical Center, Seattle, WA USA; 17 Genomic Medicine Institute (M.S.W.), Geisinger, Danville, PA USA; 18 National Human Genome Research Institute, National Institutes of Health, Bethesda, MD USA; 19 U.S. Department of Veterans Affairs Medical Center, Cincinnati, OH USA

**Keywords:** Development, Obesity

## Abstract

**Background/Objectives:**

Melanocortin-4 receptor (MC4R) plays an essential role in food intake and energy homeostasis. More than 170 *MC4R* variants have been described over the past two decades, with conflicting reports regarding the prevalence and phenotypic effects of these variants in diverse cohorts. To determine the frequency of *MC4R* variants in large cohort of different ancestries, we evaluated the *MC4R* coding region for 20,537 eMERGE participants with sequencing data plus additional 77,454 independent individuals with genome-wide genotyping data at this locus.

**Subjects/Methods:**

The sequencing data were obtained from the eMERGE phase III study, in which multisample variant call format calls have been generated, curated, and annotated. In addition to penetrance estimation using body mass index (BMI) as a binary outcome, GWAS and PheWAS were performed using median BMI in linear regression analyses. All results were adjusted for principal components, age, sex, and sites of genotyping.

**Results:**

Targeted sequencing data of *MC4R* revealed 125 coding variants in 1839 eMERGE participants including 30 unreported coding variants that were predicted to be functionally damaging. Highly penetrant unreported variants included (L325I, E308K, D298N, S270F, F261L, T248A, D111V, and Y80F) in which seven participants had obesity class III defined as BMI ≥ 40 kg/m^2^. In GWAS analysis, in addition to known risk haplotype upstream of *MC4R* (best variant rs6567160 (*P* = 5.36 × 10^−25^, Beta = 0.37), a novel rare haplotype was detected which was protective against obesity and encompassed the V103I variant with known gain-of-function properties (*P* = 6.23 × 10^−08^, Beta = −0.62). PheWAS analyses extended this protective effect of V103I to type 2 diabetes, diabetic nephropathy, and chronic renal failure independent of BMI.

**Conclusions:**

*MC4R* screening in a large eMERGE cohort confirmed many previous findings, extend the *MC4R* pleotropic effects, and discovered additional *MC4R* rare alleles that probably contribute to obesity.

## Introduction

The melanocortin-4 receptor (*MC4R*, OMIM:155541) is a G protein-coupled receptor that is critical in leptin–melanocortin pathway. The protein MC4R is predominantly expressed in the hypothalamus and is involved in regulation of satiety, feeding behavior, and energy homeostasis [1, 2]. *MC4R*-knockout mice are hyperphagic with a reduced metabolic rate and elevated body weight [3] with body mass increased by 7–45% in heterozygous and 50–100% in homozygous genotypes in comparison to wild type [4, 5]. In humans, more than 170 distinct rare variants are associated with early onset obesity and hyperphagia [6]. *MC4R*-associated obesity is the most common monogenic form of obesity with a reported prevalence of up to 6% [7]. However other reports have shown lower prevalence [8, 9]. In the last two decades, in vitro assays have been developed in order to determine the biosynthesis, cell-surface expression, ligand binding, and Gs activation (measurement of cAMP) of MC4R and its naturally occurring variants [10, 11]. However, the observed functional defects are sometimes discordant with obesity due to complex gene environmental interactions and variable expressivity of dominant inheritance [12]. Moreover, gain-of-function variants are also known. From a meta-analysis, V103I appears to be protective against obesity (OR 0.69) [13, 14]. The V103I variant may reduce receptor internalization [15]. Another missense variant I251L originally classified as functionally neutral shows increased MC4R basal activity through alteration of cAMP signal transduction that protects against obesity (OR = 0.5) [16]. Indeed, in a large United Kingdom Biobank study of 450 K individuals, 11 out of 61 studied variants had potential gain-of-function properties that await confirmation [15]. Because of this heterogeneity and that most original studies were limited to severely obese families that may overestimate the penetrance estimates, larger studies from unselected populations are needed to better elucidate and confirm prior results. Moreover, apart from rare coding variants, common noncoding regulatory variants upstream of *MC4R* gene are known determinants of BMI variation at the population level that add complexity [17–19].

The Electronic Medical Record (EMR) is a rich source of clinical information. In 2007, The electronic MEdical Records and GEnomics (eMERGE) network was initiated by the National Human Genome Research Institute (NHGRI) to explore the utility of DNA biobanks linked to EMRs for research [20]. Recently, the network developed protocols to perform genetic sequencing of 109 of the most clinically relevant genes including *MC4R* [21].

In this study, we evaluate and catalog the *MC4R* sequencing and genotyping data from eMERGE III participants to further study this genomic region and its association with obesity and related phenotypes.

## Materials/Subjects and methods

### Study cohort and sequencing

Protocols for this study were approved by the Institutional Review Boards at each institution; all included participants provided written informed consent at study enrollment.

### eMERGE-seq

The sequencing data obtained from the eMERGE III Network consist of 24,956 participants from 11 US sites. The network developed protocols to sequence 109 genes including *MC4R* [21]. Two CAP/CLIA (College of American Pathologists (CAP), Clinical Laboratory Improvement Amendments (CLIA)) certified DNA sequencing laboratories, Baylor College of Medicine Human Genome Sequencing Center and Broad Institute and Partners Laboratory for Molecular Medicine were responsible for sequencing, data harmonization and quality control (QC) analyses. Details of these procedures have been reported previously [21].

### eMERGE-GWAS

Apart from sequencing data, postimputation whole-genome genotyping data for additional 77454 independent participants from eMERGE network were available to us (dbGAP (phs000888.v1.p1)). The imputation process and genotype QC in eMERGE followed guidelines that have been published previously [22]. The QC process included sample call rates, sample relatedness, population stratification, and sex inconsistency as well as marker quality (i.e., marker call rate, minor allele frequency (MAF), and Hardy–Weinberg equilibrium (HWE)). All common variant analyses were limited to participants with call rates ≥ 98%, variants with call rates ≥ 99%, as well as variants with MAF ≥ 1% and HWE *P* ≥ 0.00001.

### Phenotype data

Demographic and anthropomorphic measures from all participants were accessed from eMERGE coordinating center and included in Table 1(a), (b). First, available BMI (kg/m^2^) data across all sites with a total of 3,368,260 entries were investigated for missing data, lab entry errors, and data inconsistency. The algorithm and initial screening indicated that BMI values above 100 kg/m^2^ most likely were due to lab entry errors in these cohorts and therefore excluded. On average, there were 30 BMI records per participant with life span duration of 10 years. Next, the mean and median BMI per participant was calculated. The median BMI per individual was then used for common variant genetic analyses. For participants with rare *MC4R* variants, all sites were recontacted to retrieve any additional missing BMI reports, and check for data inconsistency. For this group, the highest post-QC BMI was used for penetrance estimation and we made sure that temporary physiologic conditions that influence BMI such as pregnancy, ascites, or edema were not present at the same time. BMI-for-age percentile was separately calculated for pediatric participants using CDC-based online resource (https://www.cdc.gov). After removing missing BMI data, 20,537 individuals from eMERGE-seq cohorts and 77,454 from eMERGE-GWAS were used for the analyses. As shown in Table 1(a), the mean age of adults and pediatrics were 58.47 and 11.73, respectively, (Table 1(a)).

**Table 1:** (**a**) The demographic distribution of EMR-linked eMERGE cohorts. (**b**) BMI breakdown by age, sex, cohorts, known race, as well as ethnicity.

(a)			
Total	Age	Female/Male	BMI—female/BMI—male^b^
Adult	58.47 (SD = 15.98)	43904/32657	29.15 (SD = 7.35)/29.35 (SD = 5.70)
Pediatrics^a^	11.73 (SD = 5.92)	9547/11883	22.02 (SD = 7.36)/20.65 (SD = 6.34)

(b)								
	Race						Ethnicity	
	EA		AA		Asian		Hispanic	
	Female	Male	Female	Male	Female	Male	Female	Male
Adult								
eMERGE-seq								
Age	*N* = 6654, mean = 51.46, SD = 15.72	*N* = 4771, mean = 56.58, SD = 14.50	*N* = 710, mean = 49.48, SD = 17.32	*N* = 372, mean = 51.02, SD = 16.76	*N* = 855, mean = 47.81, SD = 11.86	*N* = 492, mean = 50.61, SD = 12.02	*N* = 664, mean = 47.60, SD = 15.55	*N* = 344, mean = 49.74, SD = 14.95
BMI^b^	Mean = 29.34, SD = 7.15	Mean = 29.48, SD = 5.69	Mean = 32.33, SD = 8.45	Mean = 29.13, SD = 6.09	Mean = 25.10, SD = 5.37	Mean = 26.53, SD = 4.12	Mean = 29.10, SD = 6.90	Mean = 28.93, SD = 5.65
eMERGE-GWAS								
Age	*N* = 29440, mean = 59.36, SD = 16.07	*N* = 23847,mean = 63.14,SD = 14.26	*N* = 3707, mean = 50.46, SD = 15.57	*N* = 1555, mean = 54.31, SD = 14.64	*N* = 352, mean = 51.47, SD = 17.26	*N* = 230, mean = 54.07,SD = 17.26	*N* = 1130, mean = 49.73, SD = 17.09	*N* = 546, mean = 54.08, SD = 16.81
BMI	Mean = 28.74, SD = 7.14	Mean = 29.40, SD = 5.65	Mean = 32.65, SD = 8.43	Mean = 29.74, SD = 6.71	Mean = 24.58, SD = 5.18	Mean = 26.27, SD = 4.35	Mean = 30.27, SD = 6.85	Mean = 29.45, SD = 5.58
Pediatrics								
eMERGE-seq								
Age	*N* = 1484, mean = 15.05, SD = 4.40	*N* = 2008, mean = 13.41, SD = 4.90	*N* = 834, mean = 12.33, SD = 5.84	*N* = 1339, mean = 11.24, SD = 6.14	*N* = 44, mean = 14.35, SD = 6.02	*N* = 50,mean = 12.69, SD = 6.42	*N* = 95, mean = 14.07, SD = 5.82	*N* = 127, mean = 12.32, SD = 5.62
BMI	Mean = 22.88, SD = 6.56	Mean = 20.93, SD = 6.07	Mean = 23.76, SD = 9.65	Mean = 20.98, SD = 7.44	Mean = 20.94, SD = 4.65	Mean = 20.74, SD = 4.30	Mean = 23.96, SD = 7.62	Mean = 22.47, SD = 7.07
eMERGE-GWAS								
Age	*N* = 4098, mean = 12.01, SD = 5.86	*N* = 5189, mean = 10.83, SD = 5.91	*N* = 2486, mean = 11.81, SD = 6.02	*N* = 2559,mean = 10.47, SD = 6.16	*N* = 115,mean = 9.51,SD = 6.61	*N* = 117, mean = 9.13, SD = 5.96	*N* = 225, mean = 12.65, SD = 5.70	*N* = 280, mean = 9.96, SD = 5.75
BMI	Mean = 21.10, SD = 6.80	Mean = 20.41, SD = 6.19	Mean = 22.60, SD = 7.65	Mean = 20.98, SD = 6.43	Mean = 18.24, SD = 4.09	Mean = 18.44, SD = 4.35	Mean = 22.89, SD = 7.57	Mean = 21.28, SD = 6.78

*BMI* body mass index (kg/m^2^), *SD* standard deviation, *EA* European American, *AA* African American, *eMERGE-seq* participants from eMERGE network with MC4Rsequencing data, *eMERGE-GWAS* participants from network with whole-genome genotyping data.

^a^Defined as ≤21 years old.

^b^Overall mean and standard deviation (SD) of precalculated median body mass index (BMI) for each individual.

### Penetrance

Since, the eMERGE participants were not preselected for obesity, population allele frequency information was used to estimate penetrance as described previously (URL: http://cardiodb.org/allelefrequencyapp/) [23]. The penetrance was defined as case allele frequency divided by control allele frequency multiplied by disease prevalence with a penetrance range of 0–1. We used traditional binary classifications to define obesity (BMI ≥ 30, pediatrics BMI ≥ 95%) and overweight (BMI ≥ 25, pediatrics BMI percentile ≥ 85%) groups, respectively. The overall prevalence of obesity in eMERGE participants was 30% consistent with US data used for penetrance estimation [24].

### Annotation and genetic analysis

All detected coding *MC4R* variants were annotated using latest version of variant annotation program (Annovar) [25]. Annotations per variant include latest ClinVar reports, evidence of previous publications in connection with obesity, exonic function, conservative prediction algorithm scores of SIFT, PolyPhen-2, MutationTaster, and PROVEAN [25]. The corresponding allele frequency per ancestry and all together for the Exome Aggregation Consortium (ExAC) and The Genome Aggregation Database (gnomAD) were also cataloged for comparison.

### GWAS analyses

For evaluation of common variants surrounding *MC4R* and genome-wide association of BMI, quantitative linear regression analyses were performed adjusting for site of genotyping (11 sites), principal components derived from genomic data (ten PCs), age, and sex using second generation of PLINK [26]. Variants with MAF above 1% that overlapped with eMERGE-GWAS successfully merged and re-imputed and only variants with high imputation info score (*r*^2^ quality score > 0.7) were selected for GWAS analyses. The HAPMAP (haplotype map of the human genome) reference population frequency and linkage disequilibrium (LD) in different ancestries were obtained using LocusZoom (http://csg.sph.umich.edu/locuszoom) and LD statistics (*r*^2^) between a pair of SNPs was calculated using PLINK [26].

At the next step, selected GWAS hits were functionally evaluated using FUMA (Functional Mapping and Annotation of Genome-Wide Association Studies) and Haplo-R [27, 28]. For the unreported variants in this study we also evaluate the effect of mutation on protein stability using STRUM, a machine learning-based mutation stability predictor algorithm [29]. This new algorithm measures the unfolding free energy difference between the wild type and mutant protein (ΔΔG) (https://zhanglab.ccmb.med.umich.edu/STRUM) [29]. In general, ΔΔG values below zero means that the mutation contributes to protein destabilization.

### Phenome-Wide Association Study (PheWAS) analyses

A PheWAS was also performed in order to evaluate pleotropic effects of variants in *MC4R* region with any other trait. The detail of methodology is described in previous publications [30]. We used the PheWAS package in R version 3.5.1 [30]. The trait definition in PheWAS approach is based on billing International Classification of Diseases (ICD) codes. ICD9 codes are collapsed into PheWAS codes (phecodes) according to the PheWAS map; then cases and controls are determined according to the code under study. We used a threshold of at least 20 cases for the code to be included in the model to have sufficient power for 1789 phecodes. Next, for each PheWAS code, a logistic regression model was created and adjusted for age, sex, and PCs. A false discovery rate (FDR) of 0.05 using the Benjamini–Hochberg method was then used to correct the threshold for multiple hypotheses testing.

### Burden test approach

In the eMERGE-seq population, PheWAS analysis was performed on extremely rare variants (MAF < 0.1%) using burden test (SNP-set (sequence) kernel association test (SKAT-O)) procedure in R, which aggregates individual score test statistics of variants while adjusting for covariates [31]. To avoid extreme case-control ratios, our criteria include subgroup of ICD9 code of ≥20 sample size in which ≥2 *MC4R* carriers, regardless of variant, were present for each trait code. We excluded the four frequent *MC4R* coding variants with reported protective or no effect (V103I, I251L, F202L, I198I). ICD9 codes related to injuries or poisoning were removed leaving a total of 1967 ICD9 codes. In this exploratory analysis, statistical significance was determined using the Bonferroni correction in which 1967 ICD9 codes remained for analyses with a *P* value threshold of (0.05/1967 = 2.55 × 10^−5^) for a single gene set.

### Power analyses

QUANTO software was used for power estimate of individual variants [32]. In GWAS analyses using BMI in linear regression model, given this small genomic region with only two independent haplotypes and large sample size (*N* = 97,991) we had >90% power to detect associations for rare variants (MAF ≥ 0.01 at Beta ≥ 0.5) and 99% power for common variants (MAF ≥ 0.2 at Beta ≥ 0.3) in an additive model. In PheWAS analyses for the binary outcomes in logistic regression, we calculated power for each variant-phecode pair given type 1 error rate of (*α* = 0.05/1789 × (2 haplotypes) = 1.39 × 10^−5^). We had more than 80% power for all reported phenotypes with MAF > 1%. PheWAS of extreme rare variants, using SKAT, was considered exploratory due to limited statistical power (12–30% power) and we reported all findings with the Bonferroni correction mentioned above in Supplementary Table S11.

## Results

After removing individuals with missing BMI measurements, this study included 20,537 eMERGE-seq participants with sequencing data for *MC4R* and additional 77,454 independent participants with postimputed genotyping data from eMERGE-GWAS. The demographic and detail of BMI distribution per race ethnicity and sex after QC are shown in Table 1(a), (b). The median BMI histogram plot for all participants is shown in Supplementary Fig. S1, which closely matched statistics reports in the United States (overall mean = 27.50, SD = 7.49) [24].

### Sequencing results

Sequencing data of *MC4R* revealed 125 variants among 1839 eMERGE-seq participants (out of the possible 24,956 sequenced, 7.3%). From these, 20,537 had post-QC BMI data. The demographic distribution of these participants as well as the frequency of BMI ≥ 30 (obesity) and BMI ≥ 25 (overweight) per ancestry are included in Supplementary Table S1. The detected variants in *MC4R* include 85 nonsynonymous, 28 synonymous, two frameshifts, two start-loss, five stop-gains, one at 3′ UTR, and two in 5′ UTR region. Sixty two of these have been previously reported or studied in connection with obesity. A comprehensive annotated overview of all variants is included in Supplementary Table S2. Overall, the allele frequencies of all variants of study participants were comparable to the reported public resources per ancestry such as ExAC and gnomAD as shown Supplementary Table S2. For clarity, we classified these variants into the three major groups:

### Group 1: pathogenic or likely pathogenic variants according to ClinVar classification

We identified 17 variants in this category for 74 *MC4R* carrier participants; 39 with BMI ≥ 30 (60%), 61 with BMI ≥ 25 (94%), and 9 with missing BMI (Supplementary Table S3). As shown, highly penetrant variant for obesity (BMI ≥ 30) include I269N (2 out of 2: meaning 2 participants had BMI ≥ 30 out of possible 2 carrier individuals), P299H (3 out of 3), I170V (7 out of 12) (pediatrics (5 out of 5)), R156Q (2 out of 2), and Q156X (3 out of 4). Consistent with prior reports, the pathogenic Y35X stop-gain variant was in complete LD with the D37V substitution for which we detected seven compound heterozygotes [33]. As shown, four had obesity and all seven were overweight (BMI ≥ 25). Apart from obesity, three of the seven had lipid disorders, the most frequent shared diagnosis (ICD9 272.4). Of note, one participant was a triple heterozygote (D37V-Y35*-I69K) with obesity (BMI = 33.1) and hyperlipidemia. The list of all compound heterozygotes and estimated penetrance per ancestry are in Supplementary Table S6.

### Group 2: Undetermined, conflicting according to ClinVar, but previously linked to obesity in at least one published study

This group consists of 45 variants (2 synonymous, 42 nonsynonymous, and 1 frameshift deletion) in which four variants had allele frequencies above 0.1% (Supplementary Table S4). The detected four frequently observed variants in *MC4R* coding region include three nonsynonymous variants of V103I (MAF = 1.6%), I251L (MAF = 0.8%), F202L (MAF = 0.1%), and one synonymous variant I198I (MAF = 0.6%). Of note, the frequency of synonymous coding variant I198I in AA was 3.7% comparable to public estimates (ExAC African = 3.4%, gnomeAD_African = 4.1%, (Supplementary Table S4)). In addition, consistent with prior reports, the F202L variant was exclusively present in combination with I198I disregarding race in which we identified 90 compound heterozygote participants (Supplementary Table S6). No significant difference in frequency of obesity between I198I-only group and I198I-F202L compound heterozygote subgroup were observed. However, we found two exceptional cases: one African American individual who was a double homozygote for I198I-F202L, an 18-year-old male with BMI of 40.2 kg/m^2^ or 99.6 percentile who also carried the diagnosis of chronic asthma. Another 2.5-year-old African American male with congenital nystagmus was a triple heterozygote for I198I-F202L-I251L with BMI in the 95th percentile.

In this group, highly penetrant variants for obesity include G252S (3 out of 3), N240S (10 out of 17), L211fs (2 out of 2), S127L (9 out of 12), and S36T (2 out of 2) (see Supplementary Table S4).

### Group 3: previously unreported variants

We identified 30 variants (26 nonsynonymous, 1 frameshift, 2 start-loss, and 1 stop-gain) that to our knowledge have not been previously linked to obesity and are predicted to result in loss of function by at least one functional algorithm or result in a frameshift with premature truncation (Table 2(a)). In addition, protein stability scores (ΔΔG) were negative for most of these variants indicating destabilization (Table 2(a)). Two additional nonsynonymous variants were identified, predicted as tolerant and listed in Table 2(a) with obesity (L325I, A114V) and a negative ΔΔG. As shown in Supplementary Table S2, some of these unreported variants previously were detected in ExAC or gnomeAD databases, but to our knowledge were not previously linked to obesity in any publication. Overall, these unreported variants were observed in 36 participants. Table 2(b) shows the frequency of obesity (BMI ≥ 30) or overweight BMI ≥ 25 condition as an outcome per ancestry and with estimated penetrance for these unreported variants. From these 36 carriers, one participant had a missing BMI; 19 were obese (BMI ≥ 30) (54%) and 28 were overweight (BMI ≥ 25) (80%). Table 3 shows the demographic and anthropomorphic measures of those with BMI ≥ 30. Highly penetrant variants for obesity (BMI ≥ 30) include (L325I (2 out of 2), and S270F (2 out of 2). Importantly, seven adult participants carrying rare variants of L325I, E308K, D298N, F261L, T248A, D111V, and Y80F had obesity class III defined as BMI ≥ 40 kg/m^2^ (Table 3). Unreported nonsense variants include the stop-gain variant Q115X that was detected in one Asian female carrier (BMI = 28.6) as well as two start-loss variants (M1I, M1V) in two African American carriers with BMI of 23.6 and 27.0, respectively. More detail anthropomorphic information of these 36 participants included in Supplementary Table S5.

**Table 2:** (**a**) Annotation of newly identified variants in *MC4R* coding region. (**b**) Number of individuals harboring the new *MC4R* variants per race/ethnicity and estimated penetrance for obesity (BMI ≥ 30) and overweight (BMI ≥ 25) [23].

(a)										
CHR	BP	REF	ALT	Function	Substitution	SIFT	Polyphen-2	MutationTaster	PROVEAN	ΔΔG^a^
18	58038610	G	T	Nonsynonymous	L325I	T	B	N	N	−1.12
18	58038660	T	A	Nonsynonymous	E308V	D	D	D	D	−1.2
18	58038661	C	T	Nonsynonymous	E308K	D	D	D	D	−1.76
18	58038666	C	T	Nonsynonymous	S306N	D	D	D	N	−1.36
18	58038684	A	G	Nonsynonymous	L300P	D	D	D	D	−3.2
18	58038691	C	T	Nonsynonymous	D298N	D	D	D	D	−0.68
18	58038708	CATG	C	Inframe deletion	I291del	.	.	.	.	
18	58038768	G	C	Nonsynonymous	P272R	D	D	D	D	−1.85
18	58038774	G	A	Nonsynonymous	S270F	D	D	D	N	−0.79
18	58038802	A	G	Nonsynonymous	F261L	D	P	D	D	−0.86
18	58038805	G	C	Nonsynonymous	P260A	D	D	D	D	−1.74
18	58038841	T	C	Nonsynonymous	T248A	D	P	D	D	0.26
18	58038856	C	G	Nonsynonymous	G243R	D	D	D	D	0.02
18	58038876	C	T	Nonsynonymous	R236H	T	B	D	N	−1.89
18	58038885	C	A	Nonsynonymous	G233V	T	B	D	N	−0.43
18	58038898	G	A	Nonsynonymous	L229F	T	P	D	D	−1.37
18	58038999	A	G	Nonsynonymous	I195T	D	D	D	D	−1.57
18	58039072	T	C	Nonsynonymous	S171G	T	B	D	N	−1.4
18	58039078	T	C	Nonsynonymous	I169V	T	B	D	N	−1.47
18	58039155	A	C	Nonsynonymous	I143S	D	D	D	D	−2.5
18	58039209	A	T	Nonsynonymous	I125N	D	D	D	D	−1.65
18	58039240	G	A	Stop-gain	Q115^a^	.	.	D	.	
18	58039242	G	A	Nonsynonymous	A114V	T	B	N	N	−0.3
18	58039251	T	A	Nonsynonymous	D111V	T	B	D	N	0.67
18	58039281	G	C	Nonsynonymous	T101S	D	D	D	D	−0.12
18	58039344	T	A	Nonsynonymous	Y80F	D	D	D	D	−1.9
18	58039353	G	A	Nonsynonymous	S77L	D	D	D	D	−1.55
18	58039377	A	T	Nonsynonymous	I69K	D	D	D	D	−3.45
18	58039431	A	C	Nonsynonymous	F51C	D	P	D	D	−0.93
18	58039435	C	A	Nonsynonymous	V50L	T	D	D	N	−0.78
18	58039580	C	T	Start-loss	M1I	D	B	N	N	−1.07
18	58039582	T	C	Start-loss	M1V	D	B	N	N	−0.78

(b)									
Substitution	N carriers	EA	AA	Asian	Other	Unknown	Hispanic^b^	Penetrance^c^ (BMI ≥ 30)	Penetrance (BMI ≥ 25)
L325I	2	1	.	.	.	1	1	1 (2/2)	1 (2/2)
E308V	2	1	1	.	.	.	.	0.65 (1/2)	0.65 (1/2)
E308K	1	1	.	.	.	.	.	1 (1/1)	1 (1/1)
S306N	1	1	.	.	.	.	.	0	1 (1/1)
L300P	1	.	1	.	.	.	.	0	0
D298N	1	1	.	.	.	.	.	1 (1/1)	1 (1/1)
I291del	1	1						0	1 (1/1)
P272R	1	1	.	.	.	.	1	1 (1/1)	1 (1/1)
S270F	2	2	.	.	.	.	.	1 (2/2)	1 (2/2)
F261L	1	1	.	.	.	.	.	1 (1/1)	1 (1/1)
P260A	1	.	1	.	.	.	.	0	0
T248A	1	1	.	.	.	.	.	1 (1/1)	1 (1/1)
G243R	1	.	.	.	.	.	.	0	1 (1/1)
R236H	2	2	.	.	.	.	.	1 (1/1), 1 missing BMI	1 (1/1), 1 missing BMI
G233V	1	.	.	.	.	.	.	1 (1/1)	1 (1/1)
L229F	1	1	.	.	.	.	.	0	0
I195T	1	.	.	1	.	.	.	0	0
S171G	1	.	.	1	.	.	.	0	1 (1/1)
I169V	1	.	.	1	.	.	.	0	0
I143S	1	1	.	.	.	.	.	0	1 (1/1)
I125N	1	1	.	.	.	.	.	1 (1/1)	1 (1/1)
Q115^b^	1	.	.	1	.	.	.	0	1 (1/1)
A114V	1	1	.	.	.	.	.	0	1 (1/1)
D111V	1	1	.	.	.	.	.	1 (1/1)	1 (1/1)
T101S	1	1	.	.	.	.	.	1 (1/1)	1 (1/1)
Y80F	1	1	.	.	.	.	.	1 (1/1)	1 (1/1)
S77L	1	1	.	.	.	.	.	1 (1/1)	1 (1/1)
I69K	1	1	.	.	.	.	.	1 (1/1)	1 (1/1)
F51C	1	1	.	.	.	.	.	1 (1/1)	1 (1/1)
V50L	1	1	.	.	.	.	.	0	1 (1/1)
M1I	1	.	1	.	.	.	.	0	0
M1V	1	.	1	.	.	.	.	0	1 (1/1)

More detail annotations are included in Supplementary Table S2.

SIFT (*D* deleterious (sift ≤ 0.05), *T* tolerated (sift > 0.05)); PolyPhen (*D* probably damaging (≥0.957), *P* possibly damaging (0.453–0.956), *B* benign (≤0.452)); MutationTaster (*D* disease causing; *N* polymorphism, probably harmless); PROVEAN (*D* deleterious, *N* neutral), *ΔΔG* unfolding free energy difference between the wild type and mutant protein [25, 29], *EA* European Americans, *AA* African Americans.

^a^Unfolding free energy difference between the wild type and mutant protein (ΔΔG).

^b^Race (EA-AA-Asian-Other-Unknown) and ethnicity (Hispanic and non-Hispanic) annotated separately.

^c^The penetrance estimate using population allele frequency (see “Methods”), (*x*/*y* = number of carriers with outcome vs total carriers).

**Table 3:** Demographic and anthropomorphic information for eMERGE obese participants (BMI ≥ 30) carrying unreported MC4R coding variants.

substitution	BMI	Age	Sex	Weight (kg)	Height (cm)	Race	Hispanic
L325I	45.41	76.28	F	76.2	.	Unknown	Y
L325I	30	47.15	M	86.6	170.2	White	N
E308V	33.81	51	F	89.35	162.56	White	N
E308K	40	28.8	M	127.89	178.85	White	N
D298N	50.15	26.45	F	112.02	149.45	White	N
P272R	33.11	44.24	F	92.99	167.59	White	Y
S270F	31.26	73	M	100.24	179.07	White	N
S270F	30.67	52	F	86.18	167.64	White	N
F261L	83.47	36.05	M	238.55	169.05	White	N
T248A	42.27	56.01	M	127.89	173.95	White	N
G233V	33.01	20.71	F	91.61	166.6	White	N
I143S	30	79.5	M	95.9	180.34	White	N
I125N	39.09	54.02	F	96.15	156.84	White	N
D111V	46.55	76	F	129.82	167	White	N
T101S	30.52	59	M	97.8	179	White	N
Y80F	58.24	53.2	M	185.16	178.31	White	N
S77L	30	71.06	M	88.18	.	White	N
I69K	33.15	60	M	106.2	179	White	N
F51C	33.36	55.93	F	96.62	170.18	White	N

Full details are in Supplementary Table S5.

### GWAS analysis

**Fig. 1:**
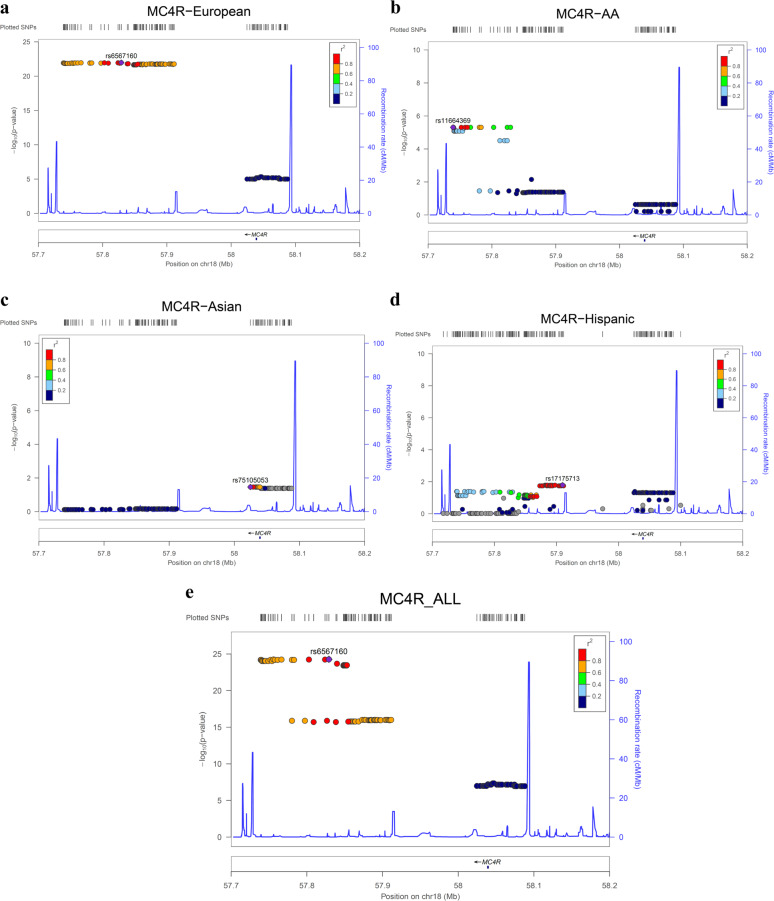
**a**–**e** LocusZoom plot of the association signals in MC4R regions for BMI with confirmation of effect at upstream of MC4R as well as identification of a novel haplotype at MC4R. Best variant rs6567160 (*P* = 5.36 × 10^−25^, Beta = 0.37). **a** GWAS effect in Europeans. **b** GWAS effect in African Americans (AA). **c** GWAS effect in Asians. **d** GWAS effect in Hispanic ethnicity. **e** GWAS signal for all ancestries. Estimated recombination rates (from HapMap) are plotted in cyan to reflect the local LD structure. The variants surrounding the most significant variant are color-coded to reflect their LD with the index variant (taken from pairwise *r*^2^ values from the HapMap database per ancestry, www.hapmap.org). Regional plots were generated using LocusZoom (http://csg.sph.umich.edu/locuszoom).

We used post-QC median BMI as a quantitative trait and performed linear regression GWAS analyses in the *MC4R* region for all eMERGE participants (*N* = 97,991) adjusted for principal components (ten PCs), age, sex, and site of genotyping. This analysis identified one common risk haplotype and another novel and relatively rare protective haplotype (Fig. 1, Supplementary Table S7). The common and known risk haplotype upstream of *MC4R* gene spans 170 KB in Europeans with the best variant rs6567160 in our study (*P* = 5.36 × 10^−25^, Beta = 0.37) (Fig. 1a, Supplementary Table S7). Other previously published variants are viable proxies (*r*^2^ ≥ 0.9) with this variant including rs571312, rs523288 rs12967135, and rs17782313. We also analyzed separately pediatric only population and report the results in Supplementary Table S7. Of note, the effect was consistent in the pediatric only population (best variant rs1942860, *P* = 2.07 × 10^−12^, Beta = 0.48, Supplementary Table S7). Ancestry specific GWAS analyses also are shown in Fig. 1a–d. Similar effect was detected in African American but at lower magnitude where the best marker was rs11664369 (*P* = 4.89 × 10^−6^, Beta = 0.51). An effect upstream of *MC4R* was not detectably significant in Asians population and was weak in Hispanic ethnicity participants (Fig. 1c, d).

In addition to this common risk haplotype, a novel rare protective haplotype spanning 63 Kb near *MC4R* was detected with no LD with the upstream common risk haplotype (Fig. 1e). This haplotype had overall frequency of 1.8% across all ancestries and encompasses the coding variant rs2229616 (V103I) (*P* = 6.23 × 10^−8^, Beta = −0.62) (Supplementary Table S7). Importantly, because of lack of LD between rs2229616 (V103I) and the main common risk variant rs6567160 (*r*^2^ = 0.0001), the protective effect remained significant after conditioning on rs6567160 (*P* = 8.77 × 10^−7^). The list of variants in this rare haplotype are shown in Supplementary Table S7; 38 intergenic markers are proxies for rs2229616 (V103I).

Next, we evaluated the potential functional effects of these variants and those included in Supplementary Table S8. Allele specific expression according to resources for brain tissue, psychENCODE, and the CommonMind Consortium indicate negative eQTL effects of the risk alleles of top markers (rs523288-T, rs6567160-C, rs11664369-T, rs17782313-C) for MC4R in brain tissue. These variants are also listed as eQTL in GTEx/v8, but no results for brain tissue are reported. In addition, markers with chromatin or histone mark regulatory effects in brain tissue according to HaploReg V4.1 includes rs2229616 (V103I) and listed in Supplementary Table S8 [28].

### PheWAS analyses

Pleotropic effects of the common and rare variants in *MC4R* region against available EMR disease traits in all eMERGE participants were evaluated by PheWAS.

For common variants, PheWAS analyses independently replicated the GWAS findings with strong association of multiple *MC4R* variants to ICD9 codes related to overweight, obesity, and morbid obesity (best *P* = 6.74 × 10^−13^) (Table 4, Supplementary Table S9). Apart from obesity, PheWAS association with common upstream variants includes dysmetabolic syndrome X, chronic venous insufficiency, and malignant neoplasm of small intestine (Table 4).

**Table 4:** PheWAS findings of all eMERGE participants at FDR < 0.05, *N* = 97,991 for two best variants in this study (rs6567160, rs2229616 (V103I)).

Description	SNP	Beta	SE	*P*	n_cases	n_controls	FDR
Obesity	rs6567160_C	0.098	0.014	8.36E−13	19253	66096	TRUE
Overweight	rs6567160_C	0.093	0.013	9.63E−13	22435	66096	TRUE
Morbid obesity	rs6567160_C	0.142	0.021	9.74E−12	6902	66096	TRUE
Type 2 diabetes	rs2229616_T	−0.291	0.051	8.48E−09	17950	67153	TRUE
Diabetes mellitus	rs2229616_T	−0.274	0.050	3.50E−08	18502	67153	TRUE
Overweight	rs2229616_T	−0.218	0.044	6.49E−07	22435	66096	TRUE
Hypertensive chronic kidney disease	rs2229616_T	−0.432	0.088	8.37E−07	6866	46505	TRUE
Morbid obesity	rs2229616_T	−0.377	0.078	1.39E−06	6902	66096	TRUE
Type 2 diabetic nephropathy	rs2229616_T	−0.573	0.120	1.71E−06	3322	67153	TRUE
Obesity	rs2229616_T	−0.221	0.047	2.08E−06	19253	66096	TRUE
Dysmetabolic syndrome X	rs6567160_C	0.221	0.048	4.00E−06	1118	89648	TRUE
Other anemias	rs2229616_T	−0.191	0.046	3.89E−05	21199	61540	TRUE
Chronic renal failure	rs2229616_T	−0.248	0.061	4.67E−05	11298	69924	TRUE
Type 2 diabetic neuropathy	rs2229616_T	−0.408	0.104	8.49E−05	3775	67153	TRUE
Nephritis; nephrosis; renal sclerosis	rs2229616_T	−0.434	0.111	8.84E−05	3685	69924	TRUE
Iron deficiency anemias NOS	rs2229616_T	−0.281	0.072	9.23E−05	7765	61540	TRUE
Hyperglyceridemia	rs6567160_C	0.140	0.036	9.80E−05	2293	47879	TRUE
Malignant neoplasm of small intestine	rs6567160_C	0.466	0.120	1.06E−04	159	86863	TRUE
Hypertension	rs2229616_T	−0.172	0.045	1.15E−04	44417	46505	TRUE
Chronic venous insufficiency	rs6567160_C	0.129	0.034	1.19E−04	2588	64412	TRUE
Bariatric surgery	rs2229616_T	−0.682	0.178	1.28E−04	1809	94483	TRUE

More details of PheWAS analyses are included in Supplementary Tables S9–S11.

Furthermore, the protective effect of V103I (rs2229616), against obesity was replicated using this approach (*P* = 1.39 × 10^−6^, Beta = −0.38). This variant also shows protective effect against type 2 diabetes, diabetic nephropathy, hypertensive nephropathy, and chronic kidney disease (Table 4). Another coding variant, the marker rs121913563 (A175T) was associated with the presence of casts and cells in urine (Supplementary Table S9). In order to avoid intercorrelated phenotypic associations that are often linked to obesity, we then reanalyzed the PheWAS controlling for BMI as another covariate. The protective effect of V103I against type 2 diabetes (*P* = 6.88 × 10^−6^) and kidney disease (*P* = 8.22 × 10^−6^) remained significant after controlling for BMI indicating multiple independent effects for this variant (Supplementary Table S10).

For extreme rare variants (MAF < 0.1%) that were limited to the eMERGE-seq population, PheWAS was performed using (SKAT-O) a burden test procedure which aggregates individual score test statistics to improve power. Abnormal glucose test (*N* = 33), colonic polyps (*N* = 29), dysmetabolic syndrome X (*N* = 11), and urinary cast (*N* = 10) were among the most frequent traits among *MC4R* carriers that while suggestive, did not achieve significance (*P* < 0.001)(Supplementary Table S11a, b). Other findings include pituitary hypofunction (*N* = 5, *P* = 1.12 × 10^−9^), neurofibromatosis type 1 (*N* = 3, *P* = 9.87 × 10^−7^), and renal dysplasia (*N* = 3, *P* = 5.52 × 10^−5^). This approach also replicated the strong link between *MC4R* and ICD9 diagnosis code of extreme BMI ≥ 70 in adult (*P* = 3.28 × 10^−34^) (Supplementary Table S11).

## Discussion

In the present study, we performed detailed annotation and penetrance estimation of 125 rare variants in *MC4R* detected in 1839 of 24,956 eMERGE participants with sequencing data. In addition, we studied 77,454 independent eMERGE participants with whole-genome genotyping data for GWAS and PheWAS of common variants in this region. *MC4R* variants represent the most frequent cause of monogenic obesity. In our collection, we found that ~7.3% of the total population and 11.3% of obese participants (BMI ≥ 30), carried at least one coding variant in *MC4R* coding region, higher than previous reports of 5 to 6% in obese patients [7] (Table 5). However, representation of coding variants varies between studies influencing the overall prevalence. Furthermore, not all rare *MC4R* variants are deleterious, hence the difference in prevalence of rare *MC4R* variants in obese and nonobese can be driven by both pathogenic and benign variants. As shown in Table 5, excluding the known V103I common coding variant with protective effect, gives the overall rate of 4.2% in our population, while increasing the fold difference of prevalence estimates especially when comparing normal vs extreme obese subgroup (OR = 7.17, 95% CI = 5.81–8.87, Table 5). Further restricting to only 17 known pathogenic variants detected in this study, yields a more than 95% overweight rate (Supplementary Table S3). Because of this heterogeneity, and other confounding factors such as the effect of race or age, we estimate penetrance per each variant in each race and age group and include in Supplementary Table S2 to contribute to future studies for collective penetrance estimations.

**Table 5:** Overall prevalence estimates (%) of MC4R coding variants across eMERGE-seq population (*N* = 24,956), with and without the common coding variant (V103I).

Outcome	*N* (%) of *MC4R* carriers (all 125 variants)	OR (95% CI)*	*N* (%) of *MC4R* carriers (excluding (V103I))	OR (95% CI)
Normal	404/8013 (5%)		208/8013 (2.6%)	
Overweight	1206/11530 (10.5%)	2.20 (1.95–2.47)^†^	789/11530 (6.8%)	2.76 (2.35–3.22)^†^
Obese	754/6655 (11.3%)	2.40 (2.12–2.72)^††^	535/6655 (8%)	3.29 (2.79–3.87)^††^
Extreme obese	254/1109 (23%)	5.59 (4.71–6.65)^†††^	178/1109 (16%)	7.17 (5.81–8.87)^†††^
All	1839/24956 (7.3%)		1069/24956 (4.2%)	

The prevalence estimates were shown for subgroups of normal ((BMI < 25, BMI% < 85%), overweight (BMI ≥ 25, BMI% ≥ 85%), obese (BMI ≥ 30, BMI% ≥ 95%), and extreme obese (BMI ≥ 40, BMI% ≥ 99%).

*An odds ratio comparison of prevalence of *MC4R* carriers for each outcome against normal population, *P* < 0.0001, (^†^overweight vs normal, ^††^obese vs normal, and ^†††^extreme obesity vs normal).

In this study, we detected 30 naturally occurring *MC4R* variants that to our knowledge have not been previously reported to be associated with obesity and are predicted to be damaging by at least one functional algorithm. These unreported variants are observed in 36 carrier individuals with 54% obesity rate. Importantly, seven adult participants carrying novel rare variants of L325I, E308K, D298N, F261L, T248A, D111V, and Y80F had obesity class III (BMI ≥ 40) and two unreported variants (L325I and S270F) were detected in two obese participants. As shown in Table 2(a), apart from predicted to be deleterious based on conservation score algorithms (SIFT and others), most of these variants also induce protein destabilization effects with negative ΔΔG scores [29].

In African Americans, the synonymous I198I variant can be seen up to 4%. In our study population, it has a frequency of 3.7% in African American in which 141 out of 242 individuals were obese (58%). Consistent with previous reports, all individuals harboring the F202L variant were compound heterozygotes with I198I. In vitro functional studies for F202L suggest both decrease or no influence on MC4R receptor activity and were observed in both obese and nonobese individuals [15, 34]. We found an exceptional double homozygote African American male (I198I-F202L) with BMI of 40.2 kg/m^2^ consistent with an allelic dosage effect. Other detected variants in African Americans include R305W, N240S, N123S, and R7H, all previously linked to obesity in the same ancestry [35].

In Hispanics, G323E previously reported in Iberians, was also detected in three Hispanics including an obese pediatric individual (Supplementary Table S2), [36]. I269N is another known pathogenic variant exclusively seen in Hispanic ethnicity with penetrance of 100% for obesity in our collection (Supplementary Table S2) [15].

Variants exclusively present in Asians include C277X, I195T, S171G, I169V, Q115X, Y35C, and R7R. C277X has been previously linked to severe obesity in the Chinese Han [37]. Similarly, we detected this variant in one adult Asian overweight male (BMI = 26.4) with hyperlipidemia (Supplementary Table S2). Y35C has been previously detected in both obese and nonobese Chinese, two of our five Asian carriers were obese (Supplementary Table S2) [38]. However, in our collection, most of Asian carriers were either lean or overweight rather than obese indicating lower penetrance of these variants in Asian or influence of other genetic or environmental factors.

Of note, in Asian populations, the WHO has recognized lower BMI cutoffs as a trigger for increased health risks [39].

In GWAS analyses, the known common risk haplotype upstream of MC4R was confirmed with the best intergenic marker rs6567160 (*P* = 5.36 × 10^−25^, Beta = 0.37). This variant as well as its proxy markers (e.g., rs17782313) near the MC4R gene have been associated with obesity in previous publications [18, 19]. To test the hypothesis that the common variants upstream of MC4R also are important determinants of BMI in children, we separately analyzed the pediatric population. The results were consistent with the adult findings with no evidence of heterogeneity (for marker rs6567160, *P* = 3.02 × 10^−12^, Beta = 0.48 (Supplementary Table S7)). Indeed, previous GWAS studies suggest a strong overlap between the genetic architecture of childhood and adult BMI [40]. This justifies our approach to combine all data together while adjusting for age and other covariates. However, we caution that BMI measures in children that are under active growth and development, even when adjusted using best methods may not be precisely comparable to adult values for the purposes of defining groups for analysis.

Interestingly we noted negative eQTL of the BMI-increasing risk alleles for *MC4R* expression in brain tissue which could functionally explain a milder form of *MC4R* deficiency for these common variants, consistent with monogenic mutation effects. To our knowledge, the only functional study for *MC4R* upstream variants was performed in nonbrain tissue (intestinal tissue) showing increased eQTL expression of *MC4R* for BMI-risk allele (rs17782313-C) [41].

PheWAS of extreme rare variants (MAF < 0.1%), using burden test methodology, provided an initial exploratory association of the *MC4R* coding variants with abnormal glucose test, presence of casts in urine, dysmetabolic syndrome X, pituitary hypofunction, and neurofibromatosis. In at least one study, signs and symptoms associated with neurofibromatosis have been reported in one case with MC4R deficiency [42]. Additional studies are needed to explore these findings in larger cohorts in order to detect true signals with sufficient statistical power.

We studied a large, unbiased, geographically diverse population, confirmed previous findings, extended associations to other related phenotypes, and added 30 new findings that warrant further evaluation in independent cohorts. The penetrance estimation for BMI and obesity from this collection will assist variant reclassification for *MC4R* and improve clinical decision making in the context of personalized medicine. These results are also relevant for targeted drug development of improved next generation melanocortin agonist therapies.

## Supplementary information

Supplementary Tables
